# Double malnutrition and associated factors in a middle-aged and older, rural South African population

**DOI:** 10.1186/s40795-024-00890-6

**Published:** 2024-06-10

**Authors:** Faheem Seedat, Stephen M. Tollman, Wayne Twine, Anne R. Cappola, Alisha N. Wade

**Affiliations:** 1 Division of Endocrinology and Metabolism, Helen Joseph Hospital, University of the Witwatersrand, Johannesburg, South Africa; 2 MRC/Wits Rural Public Health and Health Transitions Research Unit, School of Public Health, University of the Witwatersrand, Johannesburg, South Africa; 3 School of Animal, Plant and Environmental Sciences, University of the Witwatersrand, Johannesburg, South Africa; 4 Division of Endocrinology, Diabetes and Metabolism, Perelman School of Medicine, University of Pennsylvania, Philadelphia, PA USA; 5 Department of Internal Medicine, School of Clinical Medicine, University of the Witwatersrand, Johannesburg, South Africa

**Keywords:** Obesity, Anaemia, Iodine deficiency, Malnutrition, South Africa

## Abstract

**Introduction:**

Double malnutrition (co-existing overnutrition and undernutrition) is increasingly prevalent in sub-Saharan Africa due to rapid epidemiological and nutritional transitions. In this region, studies of double malnutrition have largely been conducted at country and household level, with individual-level studies primarily limited to children and women of reproductive age. We investigated the prevalence and determinants of individual-level double malnutrition in middle-aged and older adults who constitute an increasing proportion of the sub-Saharan African population.

**Methods:**

250 individuals aged 40–70 years (50% women) and resident in the Agincourt Health and socio-Demographic Surveillance System in rural Mpumalanga province, South Africa, were randomly selected. Double malnutrition was defined as overweight/obesity and anaemia only, overweight/obesity and iodine insufficiency, or overweight/obesity and any micronutrient deficiency (anaemia and/or iodine insufficiency). The Chi-squared goodness of fit test was used to compare the expected and observed numbers of individuals with the type of double malnutrition. Logistic regression was used to investigate determinants of each type of double malnutrition.

**Results:**

Double malnutrition was present in 22–36% of participants, depending on the definition used. All types of double malnutrition were more common in women than in men (overweight/obesity and anaemia: 34% vs. 10.2%, *p* < 0.01; overweight/obesity and iodine insufficiency: 32% vs. 12.2%, *p* < 0.01 and overweight/obesity and any micronutrient deficiency: 50.5% vs. 20.4%, *p* < 0.01). There were no differences between the overall expected and observed numbers of individuals with combinations of overweight and micronutrient deficiencies [overweight/obesity and anaemia (*p* = 0.28), overweight/obesity and iodine insufficiency (*p* = 0.27) or overweight/obesity and any micronutrient deficiency (*p* = 0.99)]. In models adjusted for socio-demographic factors, HIV and antiretroviral drug status, and food security or dietary diversity, men were 84–85% less likely than women to have overweight/obesity and anaemia, 65% less likely to have overweight/obesity and iodine insufficiency and 74% less likely to have overweight/obesity and any micronutrient deficiency.

**Conclusions:**

Individual-level double malnutrition is prevalent in middle-aged and older adults in a rural sub-Saharan African community. Interventions to improve nutrition in similar settings should target individuals throughout the life course and a focus on women may be warranted.

## Introduction

Increased consumption of calorie-dense, nutrient-poor foods and decreased physical activity have led to a double burden of malnutrition in lower- and middle-income countries, with co-existing undernutrition and overnutrition at country, household and individual levels [1]. Double malnutrition in adults may be defined as the co-occurrence of micronutrient deficiencies and overweight/obesity. Each of these is independently associated with increased morbidity [2–5] and they have a complex interaction, with evidence to suggest overweight/obesity may impair micronutrient metabolism [6, 7] and micronutrient deficiencies may exacerbate obesity-related cardiometabolic diseases [8–10].

In sub-Saharan Africa, many countries are undergoing rapid epidemiologic and nutritional transition, and factors such as food insecurity, HIV positivity and antiretroviral drug use, which may contribute to double malnutrition, are common [11–13]. Although existing data suggest prevalent double malnutrition at country and household levels in this region [14], less is known about individual-level double malnutrition, and the data that do exist have largely been obtained from children and women of reproductive age, where prevalence varies considerably between 2 and 40% [15–17]. There is a conspicuous lack of information on double malnutrition in middle-aged and older adults in whom both micronutrient deficiencies and overweight/obesity are associated with poorer outcomes [18, 19] and who constitute an increasing proportion of the sub-Saharan African population.

We aimed to investigate the prevalence and determinants of individual-level double malnutrition in middle-aged and older adults in a rural South African community in which population-level double malnutrition has been reported [20].

## Methods

**Fig. 1:**
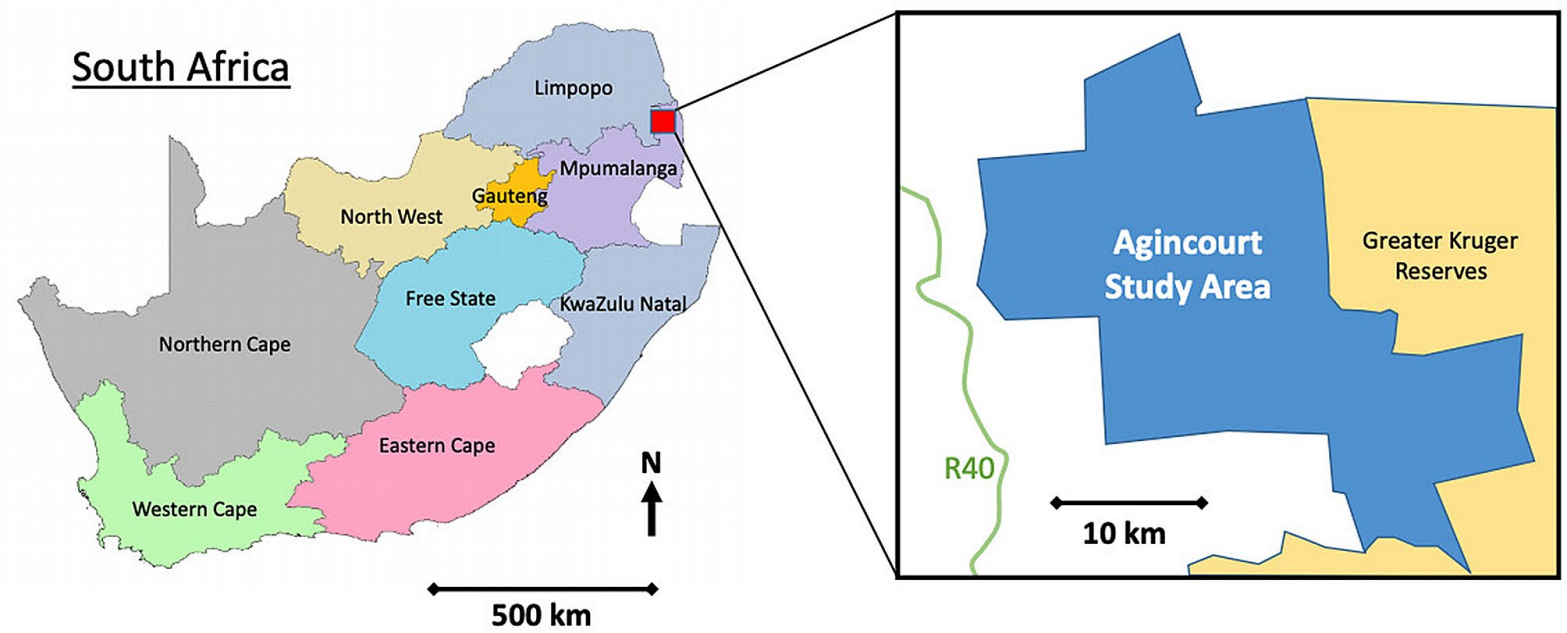
Map of South Africa showing location of Agincourt Health and Socio-Demographic Surveillance System site

Of 2,486 individuals jointly enrolled in the population-based Health and Ageing in Africa: a Longitudinal Study of an INDEPTH Community in South Africa [21] and Genomic and Environmental Risk Factors for Cardiometabolic Disease in Africans [22] cohorts, 250 individuals (50% women) were randomly selected. The sample size was based on the recommended number of spot urine samples necessary to estimate population iodine status with 95% confidence and within 10% precision [23]. Individuals were eligible for inclusion in the cohort studies if they were aged 40–70 years and were resident in the Agincourt Health and socio-Demographic Surveillance System site, situated in rural Mpumalanga province 500 km northeast of Johannesburg, South Africa (Fig. 1) [24] and participants were randomly selected from site residents. Data collection was performed by trained research staff and took place during the home and clinic visits in which all participants took part and which occurred between November 2014 and December 2016.

### Questionnaire data

Socio-demographic and food security data were self-reported, with dietary diversity and experience-based food availability (experience of food shortage and number of meals eaten) used to assess household food security [25]. Daily dietary diversity was calculated by summing the number of times that eggs, vegetables and the most commonly eaten animal protein and most commonly eaten starch were consumed in a year and then dividing by 365; diversity was then rescaled by dividing by four, with a score range of 0–1, with 0 indicating the least diversity and 1 the most diversity.

The food security score was generated by summing the response to the question “Have there been any days in the last month when your household experienced a shortage of food to eat?” (0 points for yes, 1 point for no), the number of meals eaten the previous day (maximum four and rescaled by dividing by 4) and the rescaled daily dietary diversity score. The food security score was rescaled by dividing by 3, with 0 the lowest possible score and 1 the highest. Wealth quintiles were determined using a principal components analysis of reported household assets [26].

### Physical examination data

Weight and height were measured by trained fieldworkers using standard procedures. Participants were weighed without shoes or heavy clothing, using a digital Physician Large Dial 200 kg capacity scale (Kendon Medical, South Africa) and weight was recorded to the nearest 0.1 kg. Standing height was measured with the participant without shoes or in light socks using a Harpenden digital stadiometer (Holtain, Wales) affixed to a wall and recorded to the nearest millmetre. Body mass index (BMI) was calculated as weight (kg) divided by height (m^2^).

### Biochemical data

Capillary blood samples were tested for haemoglobin at point of collection (Haemocue Hb 201 + analyser; Haemocue, Sweden). Dried blood spots were analysed for HIV serostatus using the Vironostika Uniform 11 (Biomeriuex, France) screening assay. Positive tests were confirmed with Roche Elecsys (Roche, USA). Dried blood spots which tested positive for HIV were further analysed at the University of Cape Town in South Africa for emtricitabine and lamivudine, components of standard first and second line antiretroviral regimens in South Africa. Early morning spot urine samples were collected from participants after an overnight fast and centrifuged on collection, with the supernatant frozen at -80 °C until analysis. Urinary iodine concentration was measured using the Pino modification of the Sandell-Kolthoff reaction with spectrophotometric detection [27] at the Centre of Excellence for Nutrition, North-West University in South Africa which participates in the Ensuring the Quality of Urinary Iodine Procedures standardisation programme conducted by the Centres for Disease Control and Prevention [28].

### Outcome definition

We defined double malnutrition as (a) overweight/obesity (defined as BMI ≥ 25 kg/m^2^/≥30 kg/m^2^).and anaemia (defined as haemoglobin < 12 g/dl in women and < 13 g/dl in men) [29] only, (b) overweight/obesity and iodine insufficiency (defined as urinary iodine concentration < 100 µg/l) [30] only, or (c) overweight/obesity and any micronutrient deficiency (anaemia and/or iodine insufficiency). This approach is in keeping with previous studies of double malnutrition in which deficiencies of one or more micronutrients are used as sentinel indicators in the definition of double malnutrition [31–33].

### Ethical considerations

Participants provided written informed consent for data collection in Shangaan, the local language. The Human Research Ethics Committee (Medical) of the University of the Witwatersrand (M141159, M170584), the Institutional Review Board of Harvard University (IRB18-0129) and the Mpumalanga Research and Ethics Committee (MP_201801_003) provided ethical approval for the primary data collection and this secondary analysis. The study was conducted in accordance with the principles of the Declaration of Helsinki.

### Statistical analyses

Continuous variables were not normally distributed and therefore described using medians and interquartile ranges, while categorical variables were described using frequencies and proportions. The Mann-Whitney test was used to investigate differences in continuous variables between those included in the analysis and those excluded and between sexes, given existing data that suggest sex differences in nutrition in this population [20, 34] while categorical variables were compared using Chi-squared and Fisher’s exact tests.

We calculated the expected number of individuals with a given type of double malnutrition as the product of the observed number of individuals with overweight/obesity and the observed number of individuals with the particular micronutrient deficiency (i.e. anaemia, iodine insufficiency or anaemia and/or iodine insufficiency) and used the Chi-squared goodness of fit test to compare the expected and observed number of individuals with the type of double malnutrition, based on previously reported methodology [32, 35].

Logistic regression was used to investigate associations between socio-demographic factors (age, educational attainment, marital status, household size, socio-economic status), HIV status, antiretroviral drug use and food security factors (food security score and dietary diversity) and each type of double malnutrition. As food security and dietary diversity were not independent metrics, they were entered into separate models.

We performed a complete case analysis, omitting individuals who were missing data on any of the included variables. A sensitivity analysis with pairwise deletion was also performed.

## Results

**Fig. 2:**
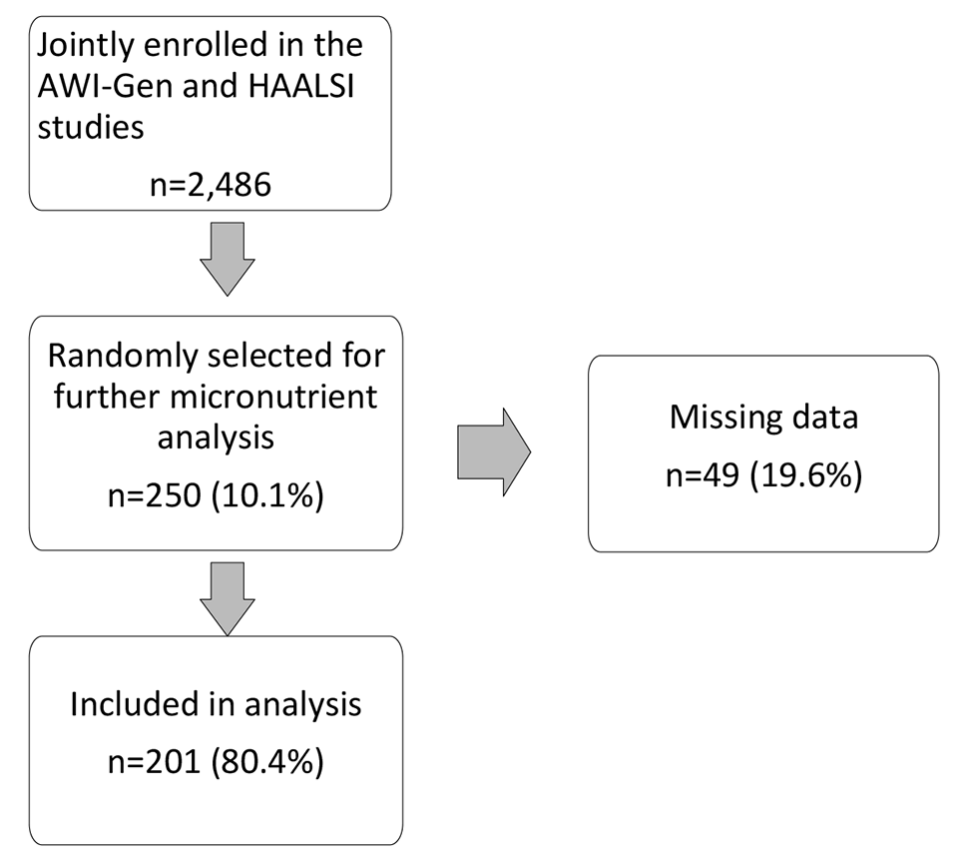
Flow chart of participant selection. AWI-Gen: Genomic and Environmental Risk Factors for Cardiometabolic Disease in Africans; HAALSI: Health and Ageing in Africa: a Longitudinal study of an INDEPTH community in South Africa

Complete data were available on 201 of the 250 randomly selected participants; these 201 individuals were then included in the analysis (Fig. 2). The median age in the 201 included participants was lower than in the 2,285 cohort members not included in the analysis (55 years [interquartile range 47–63 ] vs. 58 years [interquartile range 50–69]; *p* < 0.01). There were no differences in the sex (women 51.2% vs. 58.4%; *p* = 0.05) or socio-economic status distribution (*p* = 0.96) between the two groups.

There were no differences in median age (55 years [interquartile range 47–63] vs. 55 years [interquartile range 50–61]; *p* = 0.85), sex (women 51.2% vs. 44.9%; *p* = 0.43) or socio-economic status (*p* = 0.98) between the 201 individuals who were included in the analysis and the 49 who were excluded due to missing data.

The characteristics of the study sample are shown in Table 1. Seventy-nine individuals in the sample (39.3%) had received primary education and 142 people (70.7%) were either currently married or cohabitating. Thirty-three individuals were HIV positive, of whom 60% were receiving antiretroviral therapy, comparable to other studies in this population [13]. Food security and dietary diversity were low. With the exception of marital status (*p* < 0.01), there were no differences in socio-demographic, HIV-related, food security or dietary diversity variables between women and men.

**Table 1:** Characteristics of study sample

	Overall *n* = 201	Female *n* = 103	Male *n* = 98	*p*
Age (years)	55 (47–63)	55 (46–64)	56 (48–62)	0.93
Highest level of education				
No formal education Primary education Secondary education	73 (36.3) 79 (39.3) 39 (19.4)	38 (36.9) 42 (40.8) 20 (19.4)	35 (35.7) 37 (37.8) 19 (19.4)	
Tertiary education	10 (5.0)	3 (2.9)	7 (7.1)	0.61
Marital status				
Never married or cohabitating Currently married or cohabitating	13 (6.5) 142 (70.7)	3 (2.9) 58 (56.3)	10 (10.2) 84 (85.7)	
Previously married or cohabitating	46 (22.9)	42 (40.8)	4 (4.1)	< 0.01
Household size	7 (5–10)	7 (5–10)	7 (5–10)	0.63
Socioeconomic status quintile				
First Second Third Fourth	29 (14.4) 49 (24.4) 26 (12.9) 48 (23.9)	9 (8.7) 26 (25.2) 14 (13.6) 23 (22.3)	20 (20.4) 23 (23.5) 12 (12.2) 25 (25.5)	
Fifth	49 (24.4)	31 (30.1)	18 (18.4)	0.09
HIV positive	33 (16.4)	17 (16.5)	16 (16.3)	> 0.99
Anti-retroviral use*	18 (60.0)	7 (46.7)	11 (73.3)	0.26
Food security score	0.7 (0.7–0.8)	0.7 (0.7–0.8)	0.7 (0.7–0.8)	0.48
Dietary diversity	0.5 (0.5–0.7)	0.5 (0.5–0.7)	0.6 (0.5–0.7)	0.85

Continuous variables are presented as medians and interquartile ranges. Categorical variables are presented as frequencies and percentages. *Antiretroviral use calculated as a percentage of those who tested HIV positive

**Fig. 3:**
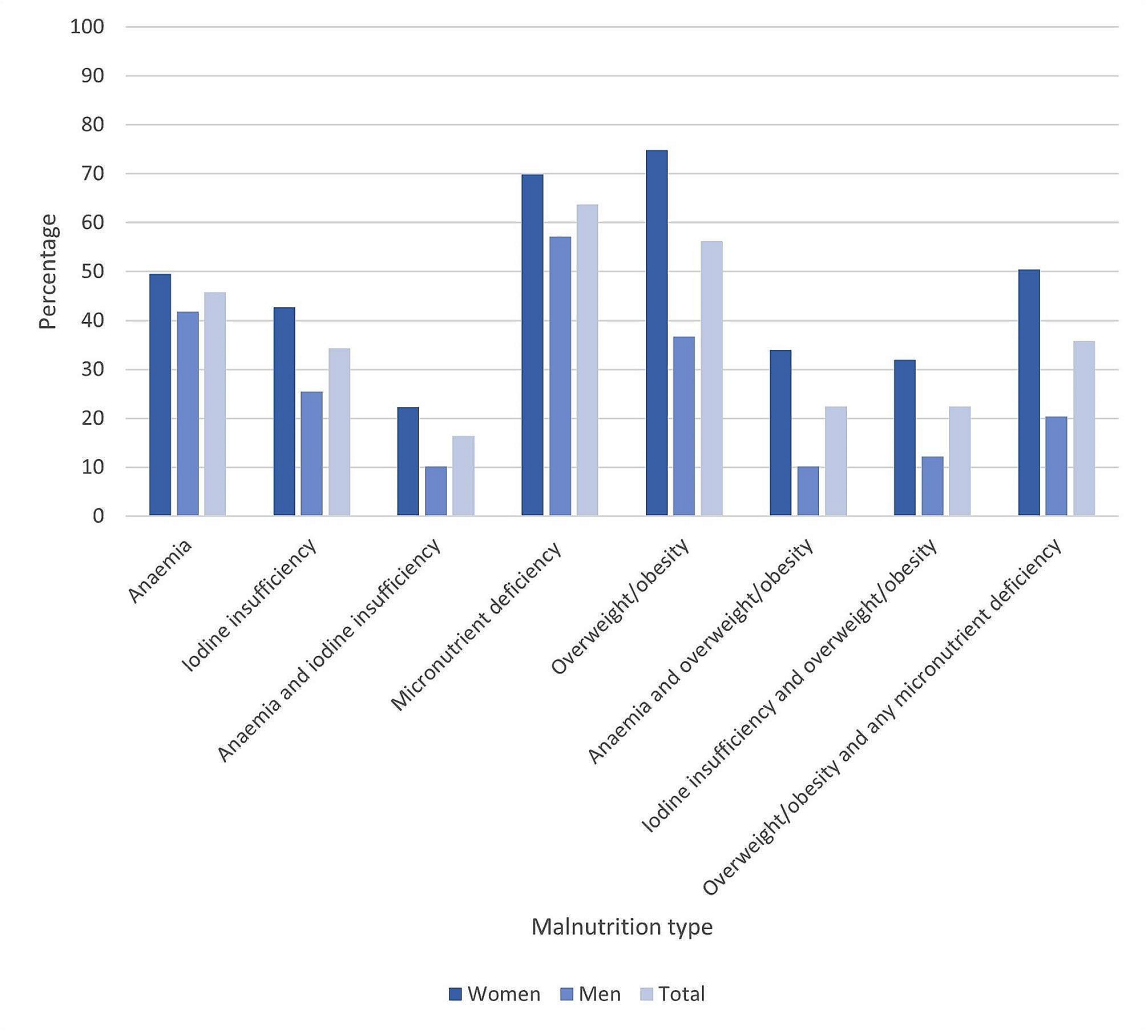
Prevalence of malnutrition in adults aged 40-70 years in a rural South African setting. Anaemia defined as haemoglobin <12 g/dl in women and <13 g/dl in men; iodine insufficiency defined as urinary iodine concentration<100 μg/l and any micronutrient deficiency defined as either anaemia or iodine insufficiency

The prevalences of various types of malnutrition are shown in Fig. 3. One hundred and twenty-eight individuals (63.7%; 95% confidence interval [CI] 56.8–70.1%) had micronutrient deficiency and 113 individuals (56.2%; 95% CI 49.2–63.0%) had overweight/obesity. Anaemia was the more common micronutrient deficiency and was present in 92 people (45.8%; 95% CI 39.0-52.7%), with 33 people (16.4%; 95% CI 11.9–22.2%) having both anaemia and iodine insufficiency. Co-occurrence of overweight/obesity and anaemia and overweight/obesity and iodine insufficiency was similar, and each present in 45 individuals (22.4%; 95% CI 17.1–28.7%), while overweight/obesity and any micronutrient deficiency was present in 72 individuals (35.8%; 95% CI 29.5–42.7%).

Two of the single forms of malnutrition, namely iodine insufficiency (42.7% [95%CI 33.4–52.5%] vs. 25.5% [95% CI 17.8–35.2%]; *p* = 0.01) and overweight/obesity (74.8% [95% CI 65.4–82.3%] vs. 36.7%; [95% CI 27.7–46.8%]; *p* < 0.01) were significantly more common in women than in men (Fig. 3) as were all types of double malnutrition (overweight/obesity and anaemia: 34% [95% CI 25.4–43.7%] vs. 10.2% [95% CI 5.5–18.1%], *p* < 0.01; overweight/obesity and iodine insufficiency: 32% [95% CI 23.7–41.7%] vs. 12.2% [95% CI 7.0-20.5%], *p* < 0.01 and overweight/obesity and any micronutrient deficiency: 50.5% [95% CI 40.8–60.1%] vs. 20.4% [95% CI 13.5–29.7%], *p* < 0.01), but there were no sex differences in the prevalence of anaemia or overall micronutrient deficiency. There were no differences between the overall expected and observed numbers of individuals with combinations of overweight and micronutrient deficiencies [overweight/obesity and anaemia (51.7 vs. 45; *p* = 0.28), overweight/obesity and iodine insufficiency (38.8 vs. 45; *p* = 0.27) or overweight/obesity and any micronutrient deficiency (72 vs.72; *p* = 0.99)]. Expected and observed numbers of individuals with each type of double malnutrition were also similar when analysed separately in women (overweight/obesity and anaemia: 53.8 vs. 52; *p* = 0.73; overweight/obesity and iodine insufficiency: 38.1 vs. 35; *p* = 0.53; overweight/obesity and any type of micronutrient deficiency: 32.9 vs. 33; *p* = 0.98) and in men (overweight/obesity and anaemia: 15 vs. 10; *p* = 0.16; overweight/obesity and iodine insufficiency: 9.2 vs. 12; *p* = 0.33; overweight/obesity and any type of micronutrient deficiency 20.6 vs. 20; *p* = 0.89).

Men were less likely than women to have any form of double malnutrition (overweight/obesity and anaemia: odds ratio [OR] 0.22, 95%CI 0.10–0.48; *p* < 0.01; overweight/obesity and iodine insufficiency: OR 0.30, 95%CI 0.14–0.62, *p* < 0.01 and overweight/obesity and any micronutrient deficiency: OR 0.25, 95%CI 0.13–0.47; *p* < 0.01) in unadjusted models. After adjustment for socio-demographic factors, HIV and antiretroviral drug status, and food security or dietary diversity, the odds of all forms of double malnutrition remained lower in men, with men 84–85% less likely than women to have overweight/obesity and anaemia (OR 0.16, 95%CI 0.06–0.44, *p* < 0.01 [food security model] and OR 0.15, 95%CI 0.06–0.42, *p* < 0.01 [dietary diversity model]) (Table 2), 65% less likely than women to have overweight/obesity and iodine insufficiency (OR 0.35, 95%CI 0.14–0.90, *p* = 0.03 [food security model] and OR 0.35, 95%CI 0.14–0.89, *p* = 0.03 [dietary diversity model]) (Table 3) and 74% less likely than women to have overweight/obesity and any micronutrient deficiency (OR 0.26, 95%CI 0.12–0.59, *p* < 0.01 [food security model] and OR 0.26, 95%CI 0.12–0.58, *p* < 0.01 [dietary diversity model]) (Table 4).

**Table 2:** Logistic regression of sociodemographic, health and food security factors associated with co-occurrence of overweight/obesity and anaemia

	Model 1			Model 2		
	OR	95% CI	*p*	OR	95% CI	*p*
Male sex	0.16	0.06–0.44	< 0.01	0.15	0.06–0.42	< 0.01
Age	1.03	0.97–1.08	0.32	1.03	0.98–1.08	0.28
Highest level of education						
No formal education Primary education Secondary education Tertiary education	ref 2.78 0.50 0.75	ref 1.12-6.87 0.12-2.01 0.07-8.08	ref 0.03 0.33 0.81	ref 2.65 0.47 0.65	ref 1.07-6.57 0.12-1.89 0.06-7.16	ref 0.04 0.29 0.72
Marital status						
Never married or cohabitating Currently married or cohabitating Previously married or cohabitating	ref 0.36 0.31	ref 0.06-2.26 0.04-2.52	ref 0.28 0.27	ref 0.37 0.29	ref 0.06-2.25 0.04-2.41	ref 0.28 0.25
Household size	0.97	0.88–1.06	0.47	0.97	0.88–1.07	0.5
Socioeconomic status quintile						
First Second Third Fourth Fifth	ref 1.64 1.75 0.64 1.31	ref 0.40-6.71 0.37-8.34 0.14-2.92 0.31-5.55	ref 0.49 0.48 0.56 0.71	ref 1.74 1.84 0.70 1.39	ref 0.42-7.17 0.39-8.79 0.15-3.25 0.33-5.94	ref 0.45 0.44 0.65 0.65
HIV positivity	0.45	0.08–2.43	0.35	0.48	0.09–2.69	0.4
Antiretroviral drug use	1.18	0.12–11.37	0.88	1.2	0.12–11.91	0.88
Food security score	7.79	0.07-914.78	0.4			
Dietary diversity				6.59	0.42-103.97	0.18

OR-odds ratio; 95%CI-95% confidence interval; Model 1 adjusted for socio-demographic variables, HIV positivity, antiretroviral drug use and food security. Model 2 adjusted for socio-demographic variables, HIV positivity, antiretroviral drug use and dietary diversity

**Table 3:** Logistic regression of sociodemographic, health and food security factors associated with co-occurrence of overweight/obesity and iodine insufficiency

	Model 1			Model 2		
	OR	95% CI	*p*	OR	95% CI	*p*
Male sex	0.35	0.14–0.90	0.03	0.35	0.14–0.89	0.03
Age	1.01	0.96–1.06	0.69	1.01	0.96–1.06	0.69
Highest level of education						
No formal education Primary education Secondary education Tertiary education	ref 2.41 1.21 3.13	ref 0.95-6.10 0.34-4.35 0.53-18.35	ref 0.06 0.77 0.21	ref 2.39 1.21 3.07	ref 0.94-6.07 0.34-4.36 0.52-17.99	ref 0.07 0.77 0.21
Marital status						
Never married or cohabitating Currently married or cohabitating Previously married or cohabitating	ref 0.29 0.65	ref 0.05-1.87 0.08-5.22	ref 0.20 0.69	ref 0.31 0.68	ref 0.05-1.94 0.09-5.39	ref 0.21 0.72
Household size	1.05	0.95–1.15	0.32	1.05	0.95–1.15	0.33
Socioeconomic status quintile						
First Second Third Fourth Fifth	ref 3.07 6.45 2.99 5.09	ref 0.53-17.73 1.02-40.79 0.51-17.72 0.87-29.74	ref 0.21 0.05 0.23 0.07	ref 3.09 6.63 3.01 5.29	ref 0.53-18.14 1.04-42.28 0.50-18.21 0.90-31.05	ref 0.21 0.05 0.23 0.06
HIV positivity	0.29	0.03–2.57	0.27	0.29	0.03–2.56	0.27
Antiretroviral drug use	2.39	0.19–30.72	0.5	2.36	0.18–30.23	0.51
Food security score	2.47	0.03-204.49	0.69			
Dietary diversity				1	0.07–14.66	> 0.99

OR-odds ratio; 95%CI-95% confidence interval; Model 1 adjusted for socio-demographic variables, HIV positivity, antiretroviral drug use and food security. Model 2 adjusted for socio-demographic variables, HIV positivity, antiretroviral drug use and dietary diversity

**Table 4:** Logistic regression of sociodemographic, health and food security factors associated with co-occurrence of overweight/obesity and any micronutrient deficiency

	Model 1			Model 2		
	OR	95% CI	*p*	OR	95% CI	*p*
Male sex	0.26	0.12–0.59	< 0.01	0.26	0.12–0.58	< 0.01
Age	1.02	0.98–1.07	0.33	1.02	0.98–1.07	0.33
Highest level of education						
No formal education Primary education Secondary education Tertiary education	ref 2.72 0.95 2.81	ref 1.21-6.13 0.32-2.83 0.55-14.41	ref 0.02 0.92 0.22	ref 2.71 0.95 2.77	ref 1.20-6.11 0.32-2.84 0.54-14.15	ref 0.02 0.92 0.22
Marital status						
Never married or cohabitating Currently married or cohabitating Previously married or cohabitating	ref 0.48 0.87	ref 0.10-2.35 0.14 − 5.40	ref 0.37 0.88	ref 0.49 0.88	ref 0.10-2.36 0.14-5.43	ref 0.38 0.89
Household size	1	0.92–1.08	0.92	1	0.92–1.08	0.92
Socioeconomic status quintile						
First Second Third Fourth Fifth	ref 1.44 3.17 1.06 1.91	ref 0.42-4.93 0.79-12.62 0.30-3.77 0.55-6.71	ref 0.74 0.09 0.85 0.42	ref 1.44 3.18 1.06 1.93	ref 0.42-4.95 0.80-12.69 0.30 − 3.80 0.55-6.75	ref 0.57 0.10 0.92 0.30
HIV positivity	0.45	0.10–1.93	0.28	0.45	0.10–1.95	0.28
Antiretroviral drug use	1.08	0.16–7.16	0.94	1.07	0.16–7.15	0.94
Food security score	1.37	0.03–58.90	0.87			
Dietary diversity				1.04	0.10-10.94	0.97

OR-odds ratio; 95%CI-95% confidence interval; Model 1 adjusted for socio-demographic variables, HIV positivity, antiretroviral drug use and food security. Model 2 adjusted for socio-demographic variables, HIV positivity, antiretroviral drug use and dietary diversity

Education was the only other factor associated with double malnutrition, with individuals with primary education twice as likely as those with no formal education to have overweight/obesity and anaemia (OR 2.78, 95%CI 1.12–6.87, *p* = 0.03 [food security model] and OR 2.65, 95%CI 1.07–6.57, *p* = 0.04 [dietary diversity model]) (Table 2) and overweight/obesity and any micronutrient deficiency (OR 2.72, 95%CI 1.21–6.13, *p* = 0.02 [food security model] and OR 2.71, 95%CI 1.20–6.11, *p* = 0.02 [dietary diversity model]) (Table 4).

Sensitivity analyses revealed similar results with double malnutrition present in 36.5% (95% CI 30.5–43%) and significantly more prevalent in women than in men (*p* < 0.01). There were no differences between the overall expected and observed number of individuals with combinations of overweight and micronutrient deficiencies [overweight/obesity and anaemia (54.8 vs. 47; *p* = 0.23), overweight/obesity and iodine insufficiency (47.8 vs. 56; *p* = 0.19) or overweight/obesity and any micronutrient deficiency (83.5 vs.84; *p* = 0.94)]. Men remained more likely than women to have any form of double malnutrition in adjusted regression models with the odds of men having overweight/obesity and anaemia identical to those in our main analysis (OR 0.16, 95%CI 0.06–0.44, *p* < 0.01 [food security model] and OR 0.15, 95%CI 0.06–0.42, *p* < 0.01 [dietary diversity model]). Men were 62% less likely than women to have overweight/obesity and iodine insufficiency (OR 0.38, 95%CI 0.16–0.91, *p* = 0.03 [food security model] and OR 0.38, 95%CI 0.16–0.91, *p* = 0.03 [dietary diversity model]) and 73–74% less likely than women to have overweight/obesity and any micronutrient deficiency (OR 0.27, 95%CI 0.12–0.58, *p* < 0.01 [food security model] and OR 0.26, 95%CI 0.12–0.58, *p* < 0.01 [dietary diversity model]) in our sensitivity analysis, estimates which were similar to our main analysis.

## Discussion

The co-occurrence of overweight/obesity and any micronutrient deficiency was present in just over a third of the individuals sampled from a middle-aged and older, rural South African population, with micronutrient deficiency more common than overweight/obesity. The observed number of individuals with double malnutrition did not differ from what would be expected by chance, suggesting that the co-occurrence of overweight/obesity and micronutrient deficiency may not be due to common pathways. Women were more likely to have any type of double malnutrition, even after controlling for socio-demographic, HIV-related and dietary factors.

We identified a higher prevalence of double malnutrition than most of the previous studies of individual-level double malnutrition in sub-Saharan Africa. Research in women aged 15–49 years in Malawi reported 10.8% had co-occurring overweight/obesity and at least one micronutrient deficiency and 3.4% had overweight/obesity and anaemia [35], while work in similarly-aged women in Ghana reported a prevalence of overweight/obesity and micronutrient deficiency of 24% [32] and the co-occurrence of overweight/obesity and anaemia varying between 6.7% and 12.4% [32, 36]. A Kenyan study which included both women and men (mean age 46 years) reported a 19% prevalence of double malnutrition [31], while in Burkina Faso, the overall prevalence of overweight/obesity and micronutrient deficiency in a sample of women and men, with a mean age of 36 years, was 8.4% [37]. These differences with our study may be due to the older age of our participants who had a median age of 55 years. Although we did not identify an association with age, our age range was rather narrow and a meta-analysis of 17 population-based studies in women of reproductive age reported higher odds of co-occurring overweight/obesity and micronutrient deficiency or co-occurring overweight/obesity and anaemia in women in the upper end of this age group when compared to younger women [33]. South Africa is also further along the epidemiologic transition than other parts of sub-Saharan Africa, which is reflected in a higher background prevalence of overweight and obesity [38].

Our finding of a higher prevalence of double malnutrition in women has not been consistently reported in other studies from sub-Saharan Africa. Zeba et al. also reported that men aged 25–60 years in urban Burkina Faso were 84% less likely than similarly-aged women to present with overweight/obesity and micronutrient deficiency compared to a phenotype without micronutrient deficiency or overweight/obesity or other cardiometabolic risk factors [37], but the prevalence of individual-level double malnutrition in Kenya was slighter higher in men (21% vs. 18%) [31] which is somewhat unexpected as the women included in that study were largely in the reproductive age group and would be expected to have a higher burden of anaemia than post-menopausal women because of menstruation and childbearing.

We did not observe a difference in the expected and observed number of individuals with double malnutrition. This is similar to previous studies which have been conducted in women of reproductive age in sub-Saharan Africa and other lower- and middle-income countries [32, 33, 35] and suggests that overweight/obesity and micronutrient deficiencies are occurring independently of each other and the sex difference in the prevalence of double malnutrition reflects the sex differences in the prevalence of its individual components. South African women are known to have significantly more overweight/obesity than men [34, 39] and while we identified a higher prevalence of iodine insufficiency in women, despite similar dietary diversity and food security, we assessed dietary diversity and food security at the household level, and it is possible than men may consume more iodine-rich foods outside of the household which was not captured in our data.

We identified higher odds of double malnutrition in individuals with primary education vs. no formal education, but did not find associations with other factors such as increasing wealth and marital status which have been identified in other lower and middle income countries [33, 35, 36], which may be due to our sample size and less variation in our explanatory varables.

Our study has several strengths. Investigations of double malnutrition in sub-Saharan Africa have largely been restricted to children, adolescents and women of reproductive age, and our study is one of the few to investigate this subject in middle-aged and older adults and men. We assessed micronutrient deficiencies using validated biochemical measures rather than self-report of dietary intake, which may be subject to recall or reporting bias, and used two indicators of micronutrient deficiency rather than one. Our study does however have limitations which must be considered. Our sample size was relatively small and our work was conducted in a rural area in South Africa which may not be representative of the country, limiting its generalisability. We also assumed anaemia was nutritional rather than due to another cause such as haemoglobinopathy or anaemia of chronic disease, Determination of anaemia is however a common method of assessing micronutrient status in population studies. Our measures of food security and dietary diversity did not inquire about calorie-dense, nutrient-poor foods or individual-level dietary habits and may therefore not have been sensitive enough to capture differences between men and women that may have contributed to our findings. Food security and diversity data may also have been subject to reporting or recall bias.

## Conclusions and recommendations

Despite these limitations, our study demonstrates prevalent double malnutrition in middle-aged and older adults in a rural sub-Saharan African population that affects women more than men and has important implications for policy makers. Although overnutrition and undernutrition appear to have different aetiologies, double duty actions that will impact both have been identified [40, 41] and these need to be targeted at individuals throughout the life course. The emphasis on the emerging epidemic of overweight and obesity in adults in sub-Saharan Africa needs to be broadened to address malnutrition more generally and a focus on women may be warranted.

Future research in this area should investigate whether our findings are reproducible in larger sample sizes in other parts of sub-Saharan Africa. Longitudinal studies are also necessary to identify whether sex differences arise de novo in middle and older ages or if, like overweight/obesity, they persist from younger ages. Additional work should also interrogate the factors responsible for the sex differences in double malnutrition to facilitate targeted interventions.

## Data Availability

The datasets analysed during the current study are available in the Harvard Centre for Population and Development Studies Dataverse at 10.7910/DVN/CXXYUU and 10.7910/DVN/F5YHML. Additional food security and urinary iodine data analysed during the current study are available from the corresponding author on reasonable request.
